# Probing the effect of aroma compounds on the hydrodynamic properties of mucin glycoproteins

**DOI:** 10.1007/s00249-020-01475-4

**Published:** 2020-11-13

**Authors:** Vlad Dinu, Thomas MacCalman, Ni Yang, Gary G. Adams, Gleb E. Yakubov, Stephen E. Harding, Ian D. Fisk

**Affiliations:** 1 National Centre for Macromolecular Hydrodynamics, School of Biosciences, University of Nottingham, Sutton Bonington Campus, Leicestershire, UK; 2 Division of Food, Nutrition and Dietetics, School of Biosciences, University of Nottingham, Sutton Bonington Campus, Leicestershire, UK; 3 International Diabetes Education and Research, Faculty of Medicine and Health Sciences, University of Nottingham, Nottingham, UK

**Keywords:** Aroma, Mucin, Interactions, Aldehydes, Phenols, Hydrodynamics

## Abstract

Aroma compounds are diverse low molecular weight organic molecules responsible for the flavour of food, medicines or cosmetics. Natural and artificial aroma compounds are manufactured and used by the industry to enhance the flavour and fragrance of products. While the low concentrations of aroma compounds present in food may leave no effect on the structural integrity of the mucosa, the effect of concentrated aroma volatiles is not well understood. At high concentrations, like those found in some flavoured products such as e-cigarettes, some aroma compounds are suggested to elicit a certain degree of change in the mucin glycoprotein network, depending on their functional group. These effects are particularly associated with carbonyl compounds such as aldehydes and ketones, but also phenols which may interact with mucin and other glycoproteins through other interaction mechanisms. This study demonstrates the formation of such interactions in vitro through the use of molecular hydrodynamics. Sedimentation velocity studies reveal that the strength of the carbonyl compound interaction is influenced by compound hydrophobicity, in which the more reactive short chain compounds show the largest increase in mucin-aroma sedimentation coefficients. By contrast, the presence of groups that increases the steric hindrance of the carbonyl group, such as ketones, produced a milder effect. The interaction effects were further demonstrated for hexanal using size exclusion chromatography light scattering (SEC-MALS) and intrinsic viscosity. In addition, phenolic aroma compounds were identified to reduce the sedimentation coefficient of mucin, which is consistent with interactions in the non-glycosylated mucin region.

## Introduction

Aroma compounds are low molecular weight compounds which are responsible for the perception of flavour. There have been over 7000 aroma compounds identified in 2014 (Parker et al. 2015). Common classes of aroma compounds include aldehydes, ketones, esters, phenols, thiols, lactones and different cyclic configurations. Fruit, vegetables, processed food and most confectionery products contain very low concentrations of aroma compounds (less than 0.01 mg/ml or 10 ppm), most of which have developed naturally during product development, such as thermal treatment or fermentation. However, some products are by design enriched with aroma compounds, to enhance their flavour. The aroma ingredients added to improve the flavour of food and other consumer products are generally recognized as safe (GRAS), and are approved for use in food, pharmaceutics and cosmetics. However, recent applications of flavour technologies led to a significant variation in the concentrations of aroma compounds in a range of products (Dinu et al. 2020). Electronic cigarettes are a prime example of a product in which the concentration of aroma compounds can exceed 5% of the entire formulation (50,000 ppm), which is approximately 10,000 times higher than the typical concentration of aroma compounds found in fruit. These concentrations are close to or even higher than it is deemed safe for consumption by the Flavor and Extract Manufacturers Association (FEMA) (FEMA FEMA, 2020a; Tierney et al. 2016; Cohen et al. 2020). Therefore, their use in e-cigarettes challenges these fundamental GRAS assumptions, because of the high concentration of aldehyde, ketone and other classes of flavourings in direct contact with the oral and respiratory mucosa.

Irrespective of the mode of administration of flavour compounds (ingestion or inhalation), their effect on the intestinal or respiratory mucosae will be determined by a similar type of interaction mechanism, varying according to the structure and function of different mucins (Linden et al. 2008). Thermodynamic and kinetic mechanisms of interaction are influenced the physical and chemical properties of each type of aroma compound (Parker et al. 2015). Most of them are highly hydrophobic and have a low solubility in water, but the shorter chain compounds are also soluble in aqueous solvents. In addition, each molecule has a different freezing and boiling point which would have a significant effect on the properties of the mucus surface, such as density and viscosity effects, but also on the properties of other biological systems in contact with the aroma compounds.

Interactions between aroma compounds and proteins have been extensively reported in the past few decades (Fares et al. 1998; Jouenne and Crouzet 2000; Paravisini and Guichard 2016; Dinu et al. 2019b). They are generally grouped into three types: (i) binding of flavour compounds, (ii) phase partitioning, i.e. air, water or lipid, or (iii) viscosity effects (Rothe 1997). Binding can either be reversible or irreversible depending on the strength of the interaction. Ketones and aldehydes were suggested to covalently bind with amino groups of proteins (Damodaran and Kinsella 1980; Weerawatanakorn et al. 2015). Others have shown that they can form weak hydrogen bonds with macromolecules containing electronegative clusters of nitrogen, sulphur or oxygen (Reineccius 2006; Tromelin et al. 2006). Although, there is no established mechanism of interaction between proteins and different classes of aroma compounds, previous studies have suggested covalent interactions between lysine and histidine residues of proteins and monounsaturated aldehydes and ketones such as hexanal, t-2-hexenal and butanone (Kikugawa et al. 1988; Meynier et al. 2004).

Mucins are the glycoprotein building blocks of the protective layer covering the mucosal membrane, lining the alimentary canal from the oral and nasal cavities through to the large intestine. They vary considerably in size, from a few thousand to several million daltons. The protein domains are rich in threonine and serine which form a bridge between their hydroxyl groups and the N-acetylgalactosamine residues of the carbohydrate region. While most mucins are heavily glycosylated (up to 90%), the ‘naked’ unglycosylated polypeptide is predominantly composed of cysteine, serine, threonine, lysine and proline which assist in the coiling of the glycoprotein which is thought to give rise to potential interaction sites with hydrophilic aroma compounds (Harding et al. 1983). These exposed amino groups are theoretically able to form several kinds of reactions with carbonyl groups, including Shiff bases or Michael addition reactions. These have also been demonstrated for more complex aldehyde compounds such as vanillin, shown to form Schiff bases with different amino acids including cysteine, lysine and phenylalanine (Kikugawa et al. 1988; Meynier et al. 2004; Ziegler 2007). In addition, reactions with cysteine were shown to be reversible under heat and acidic conditions, such as the stomach environment.

While low concentrations of aroma compounds may have negligible physical and chemical effects on the oral and intestinal mucus during oral processing, the very high concentrations of carbonyl containing flavour compounds inhaled by vaping are suggested to contribute significantly to the physical and chemical properties of the lung mucus, including viscosity effects and subsequent effects on the respiratory function. In light of this hypothesis, matrix free biophysical characterisation techniques of analytical ultracentrifugation (SV-AUC), viscometry and size exclusion chromatography multi angle laser light scattering (SEC-MALS) were employed to study some of the effects of model volatile compounds: linear aldehydes (hexanal, octanal, decanal) and linear ketones (butanone, hexanone, octanone, decanone) (Table 1). In addition, the effects of phenolic compounds (guaiacol, p-cresol and m-cresol) on the solution properties of bovine submaxillary mucin were probed using sedimentation velocity. This work along with future investigations will provide some of the starting points on the understanding some of the effects of high concentration volatile aroma compounds on the respiratory but also on the gastro-intestinal system.

**Table 1:**
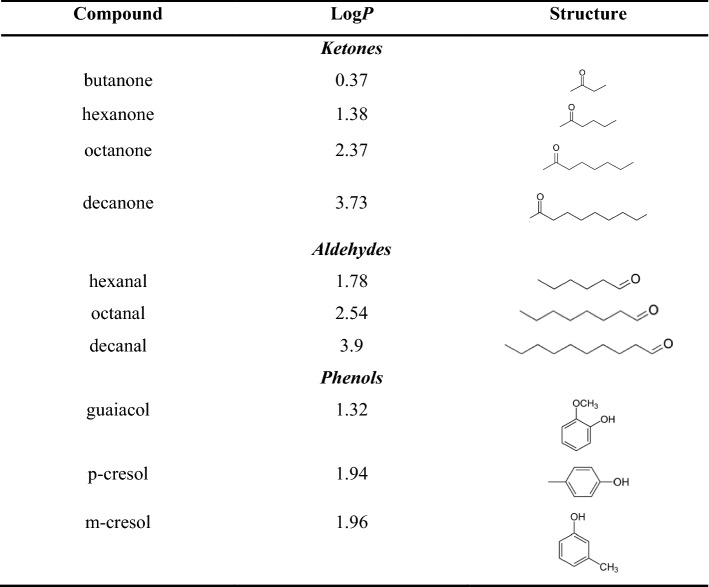
Lipophilicity (Log*P*) and chemical representations of the volatile aroma compounds used in the bovine submaxillary mucin (BSM) interaction experiments

## Materials and methods

### Sample preparation

Bovine submaxillary mucin (BSM) (type I-S, M3895) and the volatile compounds were from Sigma-Aldrich (Dorset, UK). The experiments were prepared in 0.1 M phosphate buffered saline (PBS), according to (Green 1933). Aldehydes and ketones were pre-solubilized in 70% ethanol by adding 1 mL aroma compound to 4 mL 70% ethanol to obtain a 20% (250 mg/mL stock solution). To prepare a 1 mg/mL solution of volatile compound, a 5 μL aroma compound was added to a 955 μL solution containing solubilized BSM. Phenols were added directly to their respective mucin solution.

### Analytical ultracentrifugation sedimentation velocity (SV)

The experiments were performed at 20.0 °C using the Rayleigh interference optical system in the Optima XL-I analytical ultracentrifuge (Beckman, Palo Alto, USA). A volume of 395 μL sample and 405 μl solvent, respectively, were injected into 12 mm double sector epoxy cells with sapphire windows, optically aligned to 0°as described previously (Channell et al. 2018). The samples were centrifuged at 30 000 rpm for 12 h. Raw data were analysed in SEDFIT V16.1c using the least-squares boundary modelling ls-g*(s) method, abbreviated as *g*(s) in this study, by generating sedimentation coefficient distributions, where s is the rate of particle sedimentation or sedimentation coefficient (in Svedberg units, S = 10^–13^ s). The coefficient values from the *g*(s) vs s distributions were normalised to standard conditions (viscosity and density of solvent at 20.0 °C) to give s_*20,w*_. A partial specific volume of 0.64 mL/g was employed for mucin (Dodd et al. 1998; Fisher et al. 2006). TI (time invariant) and RI (radial invariant) noise were removed during data fitting. The distributions were exported and plotted in Origin 7.5 (Origin Lab, MA, US).

### Size exclusion chromatography: multi angle light scattering (SEC-MALS)

The SEC set-up consisted of a Postnova Analysis PN7505 degassing unit (Landsberg am Lech Germany), Shimadzu LC-10AD HPLC Pump (Shimadzu UK, Milton Keynes, UK.), fitted with a Spark-Holland Marathon Basic autosampler (Spark Holland, Emmen, The Netherlands) combined with a TSK Gel guard column (7.5 × 75 mm) and TSK Gel G5000, G6000 columns (7.5 × 300 mm) connected in series (Tosoh Biosciences, Tokyo, Japan), fully flushed of column debris. Light scattering intensities were simultaneously detected at 14 angles as a function of elution volume using a DAWN^®^ HELEOS^™^ II, light scattering photometer connected in series to a ViscoStar^®^ II on-line differential viscometer, an Optilab^®^ rEX refractive index detector (Wyatt Technology Corporation, California, U.S.A.). A stock solution of 1.0 mg/mL BSM and BSM-hexanal was filtered through a 0.45 µm syringe filter (Whatman, Maidstone, England) to remove any insoluble material or dust prior to injection and then injected into the autosampler. A 100 µl aliquot of BSM and BSM-hexanal were injected onto the columns at ambient temperature (20 ± 3 °C) at a flow rate of 0.8 mL/min. ASTRA^™^ (Version 6) software (Wyatt Technology Corporation, Santa Barbara, U.S.A.) was used to estimate the weight average molecular weight, *M*w and radius of gyration *R*_g_, as a function of elution volume. The 4mW He–Ne laser was used at a wavelength of 632.8 nm, and the refractive increment used was 0.181 mL/g. Because of the low solute concentrations and column dilution, non-ideality effects were assumed negligible.

### U-tube capillary (Ostwald) viscometry

Relative viscosity was measured using the semi-automated (Schott Geräte, Hofheim, Germany) U-tube Ostwald capillary viscometer immersed in a temperature-controlled water bath at 20.00 °C. A constant volume of 2.0 mL was used for sampling BSM-hexanal mixture, automatically recorded six times. BSM concentrations were constant at 1.0 mg/mL while the buffer solution was made using different concentrations of hexanal. The intrinsic viscosity, [η] was calculated according to the Solomon–Ciuta equation (Solomon and Ciuta 2019) at a constant BSM concentration “*c*” of 1.0 mg/mL and plotted against the concentration of the solvent in Fig. 2 using:

1 $$ \left[ \eta \right] \cong \frac{1}{c}\left( {2\left( {\eta_{sp} } \right) - 2{\text{ln}}\left( {\eta_{r} } \right)} \right)^{{{\raise0.7ex\hbox{$1$} \!\mathord{\left/ {\vphantom {1 2}}\right.\kern-\nulldelimiterspace} \!\lower0.7ex\hbox{$2$}}}} . $$

## Results and discussion

### Carbonyl containing compounds

**Fig. 1:**
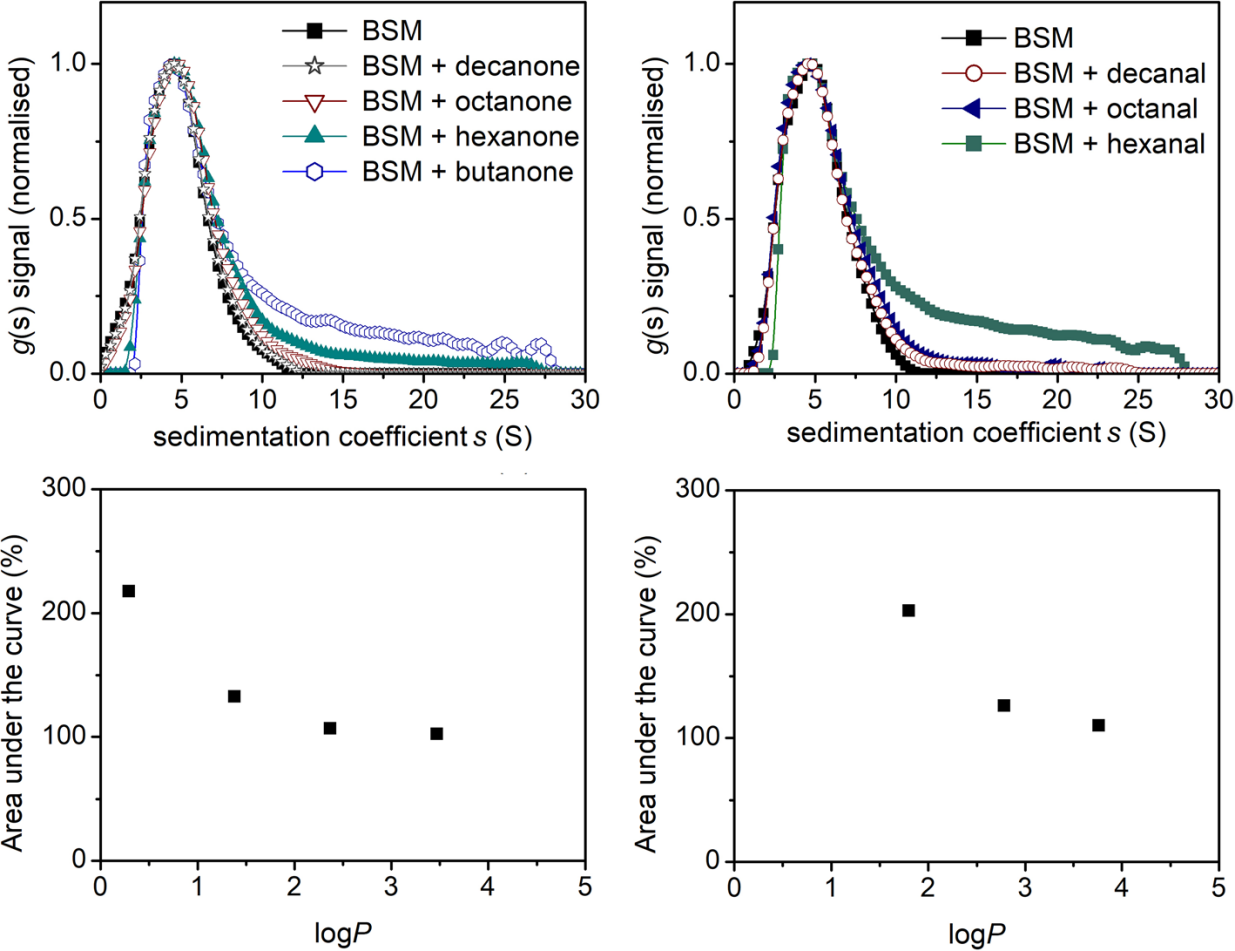
Sedimentation velocity, *g*(s) analysis showing the sedimentation coefficient distributions of bovine submaxillary mucin (1.0 mg/mL) and the effect of aldehydes and ketones (1.0 mg/mL); and bottom: plot of hydrophobicity, log*P* against % change in mucin complexation as determined by the area under the normalised sedimentation coefficient curve. Rotor speed: 30,000 rpm (90,000 g), 20.0 ºC

The sedimentation velocity results show the distribution of sedimentation coefficients for 1.0 mg/mL BSM solution, revealing a broad polydisperse distribution ranging from 2 to 12S, as shown previously (Dinu et al. 2019a). The addition of linear aldehydes and ketone volatile compounds was shown to result in an increase in the proportion of species of high sedimentation rates, tailing up to ~ 28S for the mixtures containing more hydrophilic compounds such as butanone or hexanal (Fig. 1). The effect is negatively correlated with compound hydrophobicity, appearing milder for less soluble, longer chain aldehydes but also for ketones as a whole, possibly due to the alkyl group reducing their reactivity (see Table 1). In other words, the SV data suggest that the mucin interaction with the aldehydes and ketones is stronger for the shorter chain compounds, given their higher solubility in water. This can also be evidenced directly from the raw data (Appendix Fig. 6) in which changes in the fringe concentration and shape correspond to species of higher sedimentation coefficient and higher diffusion. Although there is some sedimentation contribution from the aroma compounds, in particular hexanal and butanone, being the most soluble (Appendix Fig. 7), the signal and apparent sedimentation coefficient are too low to suggest the possibility of concomitant sedimentation with the much larger mucin components.

**Fig. 2:**
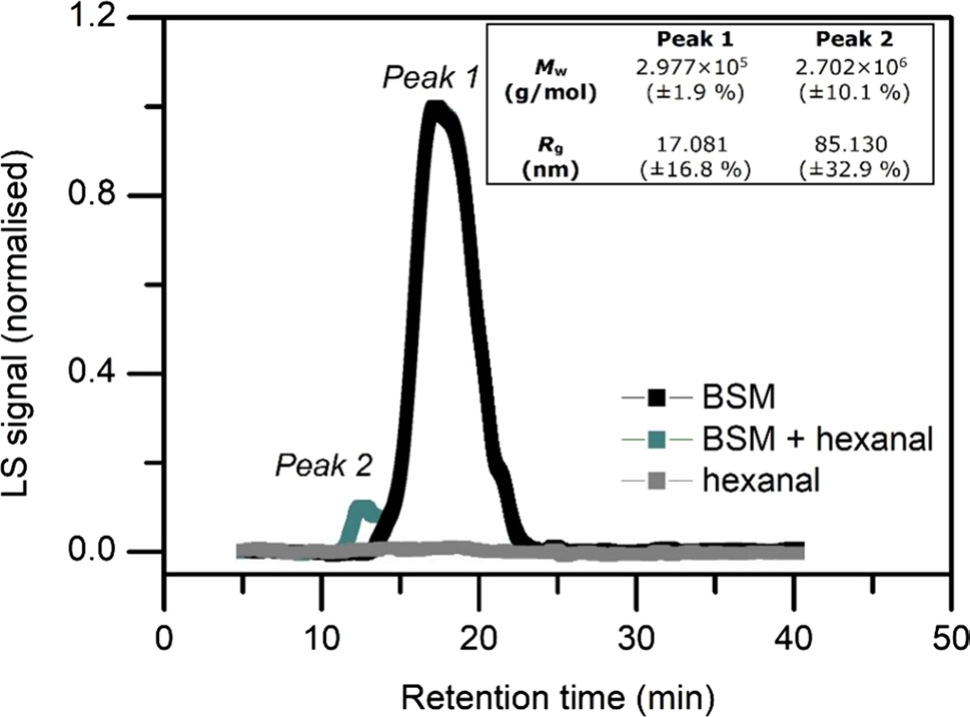
SEC-MALS results showing the light scattering (LS) elution profile of BSM, hexanal and the result of their interaction. Insert shows the summary for the hydrodynamic parameters for the main peaks, such as the apparent weight average molar mass (*M*_w_) and radius of gyration (R_g_)

The exact mechanism behind the increase in the higher S species remains unclear; however, AUC are indicative of non-specific mucin–solvent interactions causing either (i) partial mucin aggregation or (ii) changes in mucin conformation. In addition to the sedimentation results, an additional experiment was employed using the size exclusion chromatography coupled to a multi angle laser light scattering system (SEC-MALS) looking at the interaction of mucin with hexanal (Fig. 2).

During size exclusion chromatography, larger particles elute first, followed by smaller molecules. If they are too small to be detected by the column, as is the case of hexanal, they are not separated by the column and, therefore, no signal is recorded. The elution profiles (Fig. 2) reveal a broad multi-component distribution of BSM, ranging from ~ 16 to 24 min, as reported previously (Dinu et al. 2019a). However, the addition of hexanal led to the formation of larger moieties (Peak 2), which appear to be up to ten times larger than the average molecular mass for Peak 1. The apparent hydrodynamic radius *R*_g_ of the new complexes is 85 nm, which is five times larger than the average *R*_g_ values for Peak 1 although the relatively high experimental errors indicates a certain degree of size heterogeneity in addition to the limited signal resulting from its small concentration, relative to the main peak.

**Fig. 3:**
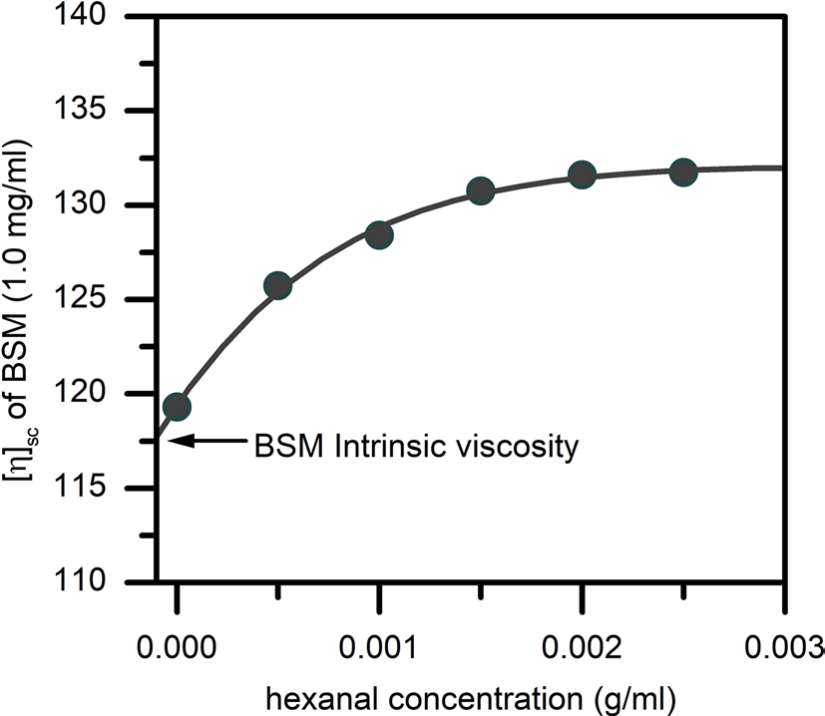
Solomon–Ciuta intrinsic viscosity [η]_sc_ analysis showing the quantitative effect of hexanal addition to bovine submaxillary mucin (1.0 mg/mL) in 0.1 M phosphate buffer saline. The polynomial fit is based on six data points derived from the separate hexanal/BSM mixtures

To further examine the effect of the aldehyde containing solvent, the intrinsic viscosity of BSM was determined in the presence of different concentrations of hexanal. They were obtained via the Solomon–Ciuta equation and plotted against concentrations of hexanal (Fig. 3). An increase in the concentrations of hexanal led to an increase in the intrinsic viscosity of mucin, gradually plateauing at higher hexanal concentrations, which is attributed to the solubility of hexanal in water (~ 2.5 mg/mL at 20.0 °C). Currently, sedimentation, size exclusion and intrinsic viscosity results confirm an interaction between volatile aldehydes/ketones and mucin. Although non-specific interactions between the glycoproteins and the solvent are suspected, due to an increase in the aggregation state of mucin, the sedimentation of the more hydrophilic aldehydes and ketones with the higher S species of the mucin is plausible, given the increase in the solute concentration (area under the curve). But the small increase in mucins intrinsic viscosity is consistent with small changes in conformation, which is suggestion to result from the binding of the carbonyls to amino regions of mucin, rather than solvent effects. A good way of confirming the complexation with the small solutes would be the use of gel chromatography, but previous attempts on mucin glycoproteins have proved unsuccessful due to smearing and inconsistent separation due to the very high molecular weight, high polydispersity and high degree of glycosylation.

### Phenolic aroma compounds

**Fig. 4:**
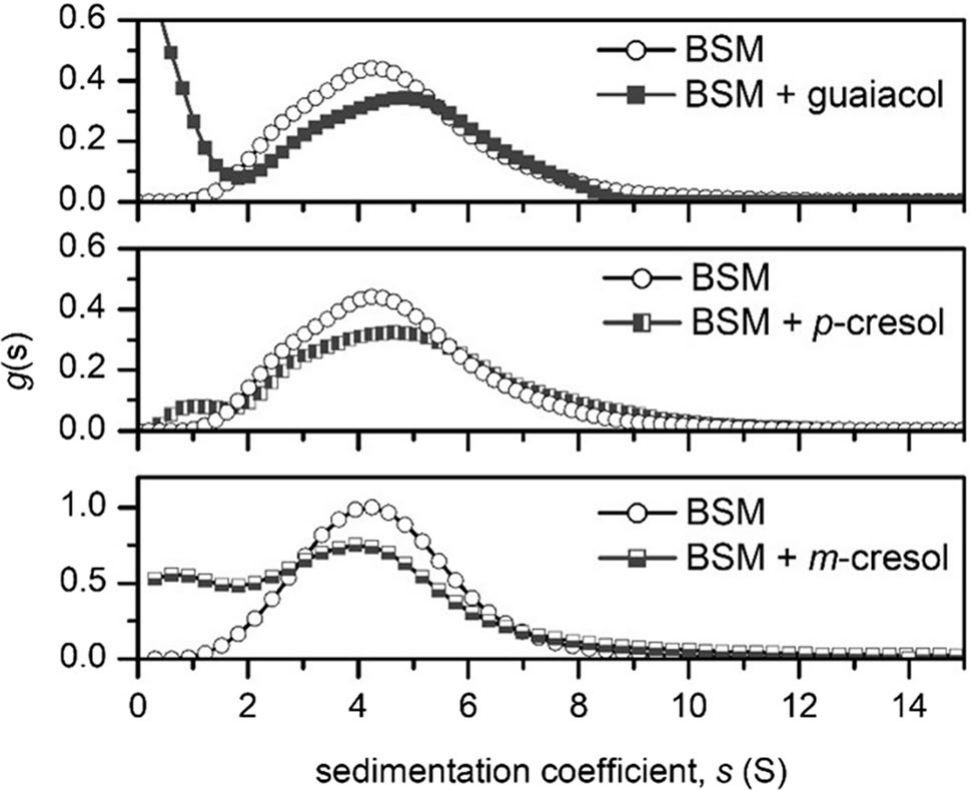
Sedimentation velocity, *g*(s) analysis showing the sedimentation coefficient distributions of BSM (0.5 g/mL) and the result of its interactions with different phenol volatile compounds (0.5 mg/mL). Rotor speed: 30,000 rpm (90,000 g), 20.0 ºC

Next, the sedimentation velocity analysis was applied to study the effects of another class of bioactive volatile aroma compounds—phenols, which have also been found to elicit changes in the sedimentation properties of mucin (Fig. 4). Although they are not directly used as food flavourings, they can be found in a range of medicines or as bi-products generated during heating of e-cigarette aerosol (Dinu et al. 2020). Changes in the sedimentation coefficient of BSM upon the addition of guaiacol, p-cresol and m-cresol suggest the formation of a smaller mucin components with a lower sedimentation coefficient, suggesting that phenols act as some type of molecular chaotropes.

These interactions were also studied by Raman spectroscopy, by examining changes in the vibrational spectrum of BSM before and after the addition of p-cresol (Appendix Fig. 8, Dinu et al. 2019a). Spectral changes were observed in the 820–850 cm^−1^ region, which represents a Fermi doublet, distinguished by the stronger (840 cm^−1^) and weaker (810 cm^−1^) Raman shifts. The ratio of the doublet is said to provide information on the strength of the hydrogen bonding of the phenoxy group in solution. Second, a decrease in the intensity of the 1500–1700 cm^−1^ Amide I and II region was observed, indicating changes in the non-glycosylated protein region of mucins (Dinu et al. 2019a).

## Discussion

Protein–aroma interactions have been reported since the 1970’s first study of protein-flavor binding (Arai et al. 1970). Using simple linear aldehydes and ketones, we aimed to gain some information on the reported interactions between flavourings which have a carbonyl functional group. SV-AUC was used to confirm that the interactions are dependent on the solubility of aldehyde and ketone flavours in water. Second, we have confirmed that aldehydes are more reactive than ketones, as shown by the increase in the concentrations and the rate of sedimenting boundaries (Appendix Fig. 6).

Other studies also confirmed a stronger affinity of aldehydes for binding of bovine serum albumin than ketones (Damodaran and Kinsella 1980). The mechanism of interaction is suggestion to arise from the binding between lysine residues and the carbonyl group of the monounsaturated aroma compounds (Kikugawa et al. 1988; Meynier et al. 2004). In addition, shorter more hydrophilic molecules such as acetaldehyde were previously demonstrated to increase the risk of developing alcoholic liver disease and hepatocellular carcinoma through a similar type of protein adduct formations, leading to impairment in protein function (Donohue et al. 1983). More recently, aroma–mucin interactions have also been proposed for more complex molecules, because there is a significant reduction in the gas phase concentration of octanal, nonanal and decanal in the presence of mucin at neutral pH, reported using gas chromatography, reinforcing the hypothesis of aroma–mucin interactions (Dinu et al. 2019b). In other studies, vanillin and ethyl vanillin which also contain additional hydroxyl and ether groups were reported to interact with DNA at the A-T groove region under physiological conditions (Qais et al. 2019). Other work also found the use of vanillin as a DNA-dependent protein kinase inhibitor for use in anti-cancer therapies (Durant and Karran 2003). While the exact mechanism remains a topic of further investigation, the current SV, viscosity and size exclusion analysis suggests that the interactions induced by the small carbonyl containing compounds are inducing changes in the conformation and/or mucin aggregation.

Unlike aldehydes and ketones, some phenol molecules such as cresols and guaiacol, the latter being a phenol with a methoxy group, were found to exhibit the opposite effect. The sedimentation experiments indicate the presence of smaller components and a decrease in the mucin components. The additional Raman experiment shows a loss in the amide region, indicative of changes in the mucin region which is free of glycosylation. Previously, phenols such as m-cresol have long been used as an excipient in insulin formulations to de-aggregate insulin and keep the proteins in their active, monomeric form (Whittingham et al. 1998). Guaiacol is another related compound which is used in the synthesis of other aroma compounds but also naturally present in the flavour of whiskey. It is used as a universal substrate for peroxidase enzymes, through the interaction with glycine and isoleucine with its phenoxy group (Murphy et al. 2012). It is worth suggesting that guaifenesin, which has the same functional groups as guaiacol, has been considered for reducing the molecular weight and viscosity of mucus in medication for patients suffering from mucus congestion issues caused by common colds (Seagrave et al. 2011). Previous work suggested that the hydroxyl group of the phenol ring (phenoxy group), is primarily involved in some type of interaction with mucin glycoproteins (Seagrave et al. 2011).

### Further remarks

**Fig. 5:**
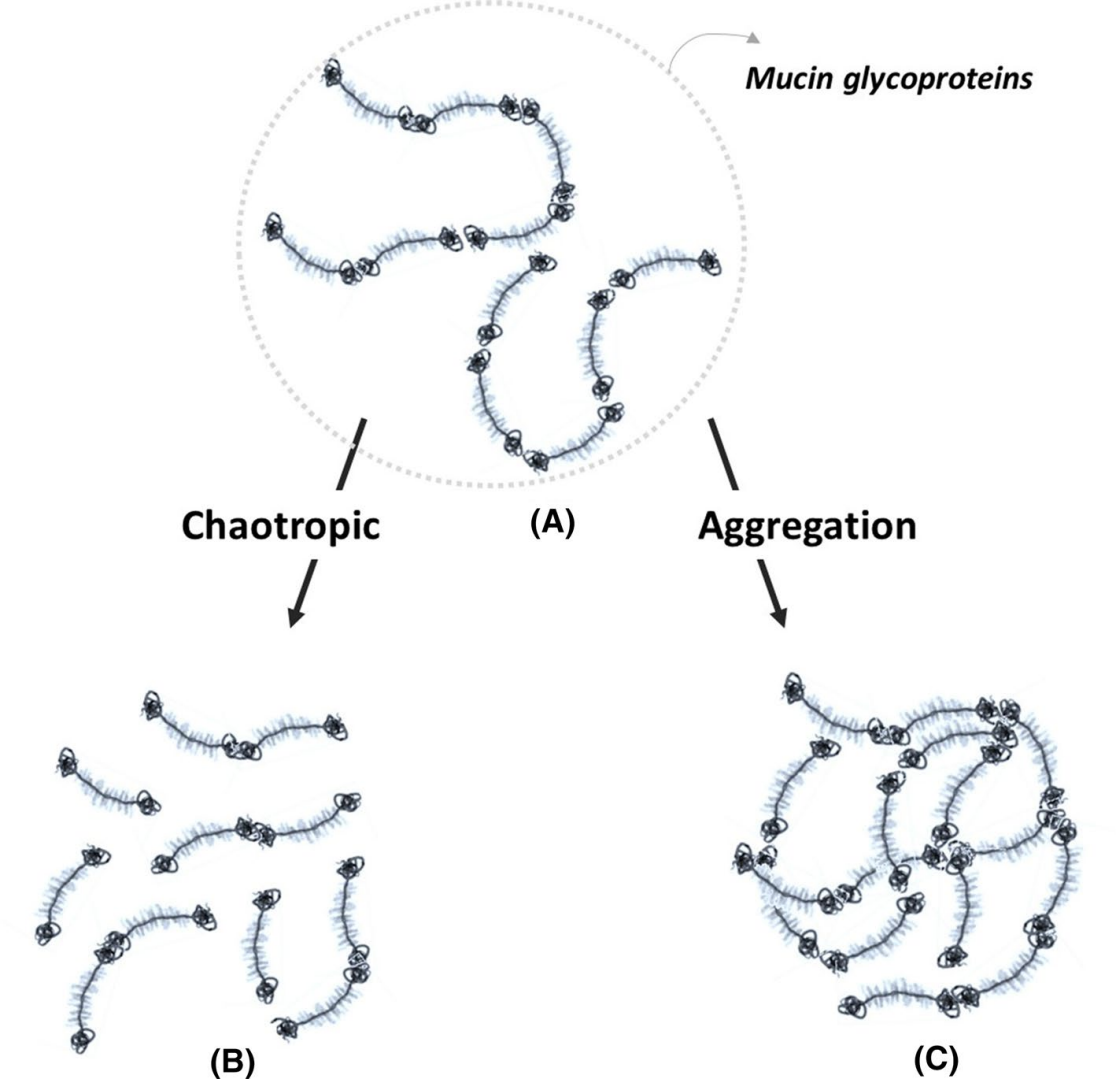
Suggested effects on mucin–mucin interactions in solution: **a** Polysdisperse, random coil model of mucin in solution showing its multimeric assembly containing the glycosylated “bottlebrush” polypeptide backbone and the non-glycosylated “naked” protein region; **b** A proposed representation of the effect of chaotropic compounds on the solution structure of mucins; **c** Proposed representation of the effects of compounds causing mucin aggregation

The exact nature by which carbonyl containing volatile compounds or the volatiles bearing a phenol group are not yet understood. Mucin glycoproteins are heavily dominated by charged oligosaccharides structures, which raise the possibility of electrostatic interactions events, such as charge shielding or charge repulsion, depending on the polarity of the volatile. Aldehydes and ketones are, therefore, suggested to have an effect on the viscoelastic properties of the mucus. In addition, volatiles bearing a phenol group are suggested to alter mucin–mucin interactions which are an important determinant in the properties of the mucus gel protecting the mucosal surfaces (Verdugo 2012). The dynamics by which the mucin stability is affected also appears to resemble that of chaotropic agents and detergents, such as urea or guanidinium hydrochloride (GuHCl), which are known to disrupt non-covalent interactions (Radicioni et al. 2016). Based on the current data and previous studies, we, therefore, suggest two possible effects on mucin–mucin interactions (Fig. 5).

While the low concentrations of volatile aroma compounds in food may not elicit a significant change on the macrostructure of mucus glycoproteins, high concentrations are, therefore, suggested to lead to a significant impairment of mucosal function. If such high concentrations are inhaled, such as vaping products, this may also be relevant to the onset cytotoxic events reported in recent literature (Tierney et al. 2016; Omaiye 2019). Although, the components used in the current study are not desirable for use in e-cigarettes, they were used to understand the effect of more complex food flavour bearing the same types of functional groups, such as aldehydes, ketones or phenols. The two effects by which aroma compounds can affect the inherent solution properties of mucins are, therefore, proposed in the current hydrodynamic study (Fig. 5).

## Conclusion

This study demonstrates the evidence to suggest aroma compounds (aldehydes, ketones, and phenols) interacts with submaxillary mucin. Based on the sedimentation results, aldehydes appear to be more reactive than ketones, with shorter chain compounds leading to species of higher sedimentation coefficients. According to size exclusion chromatography and viscosity data for hexanal, it was shown that the interactions lead to species of higher apparent molar mass, size and higher intrinsic viscosity. It is unclear whether these effects are a consequence of a direct aroma–mucin complexation induced by the interaction between carbonyls and amino groups; however, larger particle formation and conformational changes are indicated. Further investigations will continue to analyze the nature of aroma–protein interactions.

In addition, SV-AUC indicated that phenols, such as guaiacol, p-cresol, m-cresol affect the sedimentation properties of mucin as evidenced from an increase in the concentration of smaller S fractions. Complemented by Raman spectroscopy, these changes appear to be related to the observed decrease in the amide I region (~ 1640 cm^−1^), suggesting interactions are occurring in the non-glycosylated protein region of mucins. Future studies will focus on the analysis of proteins which have a well-defined structure to investigate the chemical and physical mechanisms of aroma-protein interactions. For instance, the use of the multi-wavelength detector capability could allow the screening of multiple aroma compounds with different optical properties at once, in the presence of heterogeneous mixtures of mucosal proteins and glycoproteins.

## Figures and Tables

**Fig. 6:**
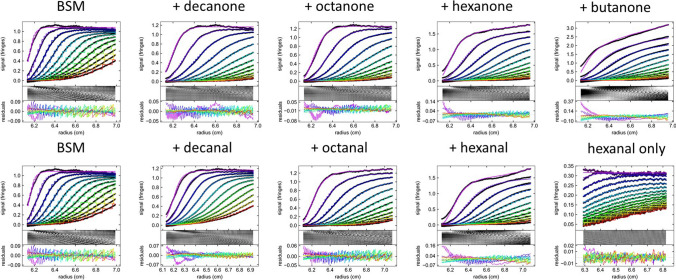
Raw data showing the changes in fringe concentration and residuals plots for BSM, BSM/aroma compounds mixtures and hexanal control, plotted using the GUSSI extension in SEDFIT (Brautigam 2015)

**Fig. 7:**
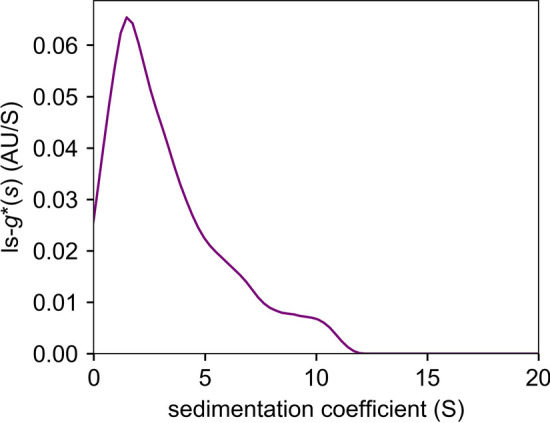
Sedimentation coefficient distribution of hexanal control (1.0 mg/mL) in 0.1 M PBS pH 7. Rotor speed: 30,000 rpm (90,000 g), 20.0 ºC

**Fig. 8:**
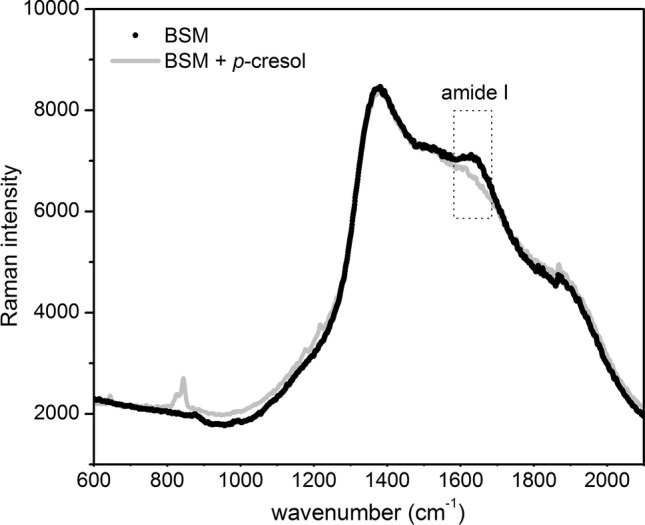
Raman spectra of BSM (c = 10 mg/mL) with and without p-cresol. The mucin/p-cresol sample was dialysed against a 14 kDa membrane prior to Raman analysis to remove excess p-cresol. Performed in 0.1 M PBS pH 7 at 20.0 °C. Adapted from (Dinu et al. 2019a)

## Data Availability

Data are available upon reasonable request.

## Appendix

See Figs. 6, 7 and 8.

## References

[CR1] Arai S, Noguchi M, Yamashita M (1970). Studies on flavor components in soybean. Agric Biol Chem.

[CR2] Brautigam CA (2015). Calculations and publication-quality illustrations for analytical ultracentrifugation data. Methods Enzymol.

[CR3] Channell G, Dinu V, Adams GG, Harding SE (2018). A simple cell-alignment protocol for sedimentation velocity analytical ultracentrifugation to complement mechanical and optical alignment procedures. Eur Biophys J.

[CR4] Cohen SM, Eisenbrand G, Fukushima S, et al (2020) GRAS 29 Flavoring Substances. https://www.ift.org/news-and-publications/food-technology-magazine/issues/2020/march/features/gras-29-flavoring-substances. Accessed 2 Jul 2020

[CR5] Damodaran S, Kinsella JE (1980). Flavor protein interactions. Binding of carbonyls to bovine serum albumin: thermodynamic and conformational effects. J Agric Food Chem.

[CR7] Dinu V, Gillis RB, MacCalman T (2019). Submaxillary mucin: its effect on aroma release from acidic drinks and new insight into the effect of aroma compounds on its macromolecular integrity. Food Biophys.

[CR8] Dinu V, Kilic A, Wang Q (2020). Policy, toxicology and physicochemical considerations on the inhalation of high concentrations of food flavour. Npj Sci Food.

[CR9] Dinu V, Yakubov G, Lim M (2019). Mucin immobilization in calcium alginate: a possible mucus mimetic tool for evaluating mucoadhesion and retention of flavour. Int J Biol Macromol.

[CR10] Dodd S, Place GA, Hall RL, Harding SE (1998). Hydrodynamic properties of mucins secreted by primary cultures of Guinea-pig tracheal epithelial cells: determination of diffusion coefficients by analytical ultracentrifugation and kinetic analysis of mucus gel hydration and dissolution. Eur Biophys J.

[CR11] Donohue TM, Tuma DJ, Sorrell MF (1983). Acetaldehyde adducts with proteins: binding of [14C]acetaldehyde to serum albumin. Arch Biochem Biophys.

[CR12] Durant S, Karran P (2003). Vanillins–a novel family of DNA-PK inhibitors. Nucleic Acids Res.

[CR13] Fares K, Landy P, Guilard R, Voilley A (1998). Physicochemical interactions between aroma compounds and milk proteins: effect of water and protein modification. J Dairy Sci.

[CR14] FEMA (2020a) Safety and regulatory authority to use flavors—focus on vaping products | FEMA. https://www.femaflavor.org/safety-regulatory-authority-use-flavors-focus-vaping-products. Accessed 2 Jul 2020

[CR15] Fisher SZ, Govindasamy L, Tu C (2006). Structure of human salivary α-amylase crystallized in a C-centered monoclinic space group. Acta Crystallograph Sect F Struct Biol Cryst Commun.

[CR16] Green AA (1933). The preparation of acetate and phosphate buffer solutions of known pH and ionic strength. J Am Chem Soc.

[CR17] Harding SE, Rowe AJ, Creeth JM (1983). Further evidence for a flexible and highly expanded spheroidal model for mucus glycoproteins in solution. Biochem J.

[CR18] Jouenne E, Crouzet J (2000). Effect of pH on retention of aroma compounds by beta-lactoglobulin. J Agric Food Chem.

[CR19] Kikugawa K, Iwata A, Beppu M (1988). Formation of cross-links and fluorescence in polylysine, soluble proteins and membrane proteins by reaction with 1-butanal. Chem Pharm Bull (Tokyo).

[CR21] Linden SK, Sutton P, Karlsson NG (2008). Mucins in the mucosal barrier to infection. Mucosal Immunol.

[CR23] Meynier A, Rampon V, Dalgalarrondo M, Genot C (2004). Hexanal and t-2-hexenal form covalent bonds with whey proteins and sodium caseinate in aqueous solution. Int Dairy J.

[CR24] Murphy EJ, Metcalfe CL, Nnamchi C (2012). Crystal structure of guaiacol and phenol bound to a heme peroxidase. FEBS J.

[CR25] Omaiye EE (2019). High concentrations of flavor chemicals are present in electronic cigarette refill fluids. Sci Rep.

[CR26] Paravisini L, Guichard E (2016) Interactions between aroma compounds and food matrix. In: Flavour. John Wiley & Sons, Ltd, pp 208–234. 10.1002/9781118929384

[CR27] Parker JK, Elmore S, Methven L (2015) Flavour development, analysis and perception in food and beverages. Elsevier. 10.1016/C2013-0-16460-4

[CR28] Qais FA, Husain FM, Khan MS (2019). Deciphering the interaction of food additive, vanillin with DNA: a biophysical and computational study. J Biomol Struct Dyn.

[CR29] Radicioni G, Cao R, Carpenter J (2016). The innate immune properties of airway mucosal surfaces are regulated by dynamic interactions between mucins and interacting proteins: the mucin interactome. Mucosal Immunol.

[CR30] Reineccius G (2006). Flavor release from foods. Flavour chemistry and technology.

[CR31] Rothe M (1997). Flavor-food interactions. ACS symposium series 633. edited by R. J. McGorrin and J. V. Leland. XII and 235 pages, numerous figures and tables. American Chemical Society, Washington, DC, 1996. Price: 89.95 US $. Food Nahr.

[CR32] Seagrave J, Albrecht H, Park YS (2011). Effect of guaifenesin on mucin production, rheology, and mucociliary transport in differentiated human airway epithelial cells. Exp Lung Res.

[CR33] Solomon OF, Ciuta IZ (2019) Détermination de la viscosité intrinsèque de solutions de polymères par une simple détermination de la viscosité—Solomon—1962—Journal of Applied Polymer Science—Wiley Online Library. https://onlinelibrary.wiley.com/doi/abs/10.1002/app.1962.070062414. Accessed 6 May 2019

[CR34] Tierney PA, Karpinski CD, Brown JE (2016). Flavour chemicals in electronic cigarette fluids. Tob Control.

[CR35] Tromelin A, Andriot I, Guichard E (2006) Protein-flavour interactions. In: Flavour in Food. Woodhead Publishing Limited, p 320. 10.1533/9781845691400.2.172

[CR36] Verdugo P (2012). Supramolecular dynamics of mucus. Cold Spring Harb Perspect Med.

[CR37] Weerawatanakorn M, Wu J-C, Pan M-H, Ho C-T (2015). Reactivity and stability of selected flavor compounds. J Food Drug Anal.

[CR38] Whittingham JL, Edwards DJ, Antson AA (1998). Interactions of phenol and m-cresol in the insulin hexamer, and their effect on the association properties of B28 pro—Asp insulin analogues. Biochemistry.

[CR39] Ziegler H (2007) Flavourings: Production, Composition, Applications, Regulations. John Wiley & Sons. 10.1002/9783527611454

